# *YUCCA9*-Mediated Auxin Biosynthesis and Polar Auxin Transport Synergistically Regulate Regeneration of Root Systems Following Root Cutting

**DOI:** 10.1093/pcp/pcx107

**Published:** 2017-09-01

**Authors:** Dongyang Xu, Jiahang Miao, Emi Yumoto, Takao Yokota, Masashi Asahina, Masaaki Watahiki

**Affiliations:** 1 Graduate School of Life Science, Hokkaido University, Sapporo, 060-0810 Japan; 2 Department of Biosciences, Teikyo University, Utsunomiya, 320-8551 Japan; 3 Faculty of Science, Hokkaido University, Sapporo, 060-0810 Japan

**Keywords:** Auxin biosynthesis, Lateral root, Polar auxin transport, Root pruning, Yucasin, *YUCCA9*

## Abstract

Recovery of the root system following physical damage is an essential issue for plant survival. An injured root system is able to regenerate by increases in lateral root (LR) number and acceleration of root growth. The horticultural technique of root pruning (root cutting) is an application of this response and is a common garden technique for controlling plant growth. Although root pruning is widely used, the molecular mechanisms underlying the subsequent changes in the root system are poorly understood. In this study, root pruning was employed as a model system to study the molecular mechanisms of root system regeneration. Notably, LR defects in wild-type plants treated with inhibitors of polar auxin transport (PAT) or in the auxin signaling mutant *auxin/indole-3-acetic acid19*/*massugu2* were recovered by root pruning. Induction of *IAA19* following root pruning indicates an enhancement of auxin signaling by root pruning. Endogenous levels of IAA increased after root pruning, and *YUCCA9* was identified as the primary gene responsible. PAT-related genes were induced after root pruning, and the YUCCA inhibitor yucasin suppressed root regeneration in PAT-related mutants. Therefore, we demonstrate the crucial role of *YUCCA9*, along with other redundant *YUCCA* family genes, in the enhancement of auxin biosynthesis following root pruning. This further enhances auxin transport and activates downstream auxin signaling genes, and thus increases LR number.

## Introduction

Organ regeneration is a distinctive feature of plants that contributes to their robustness in adverse conditions. While root system architecture is genetically determined, different environmental conditions such as water availability, nutrient levels, physical obstacles or damage modify the root system architecture (Al-Ghazi et al. 2003, Ditengou et al. 2008, Sena and Birnbaum 2010, Sugimoto et al. 2011, Van Norman et al. 2013). The plasticity of root system architecture helps plants to adapt to an ever-changing environment. The agricultural technique of root pruning is an application of root system regeneration. When part of the root system is removed by root pruning, the plants are able to regenerate a new root system with a smaller size and more branches (Wang et al. 2014). This new root system provides an efficient intensive production system with reduced vegetative growth (by reducing the flow of nutrients, water and hormones from root to shoot), which led to the promotion of solar radiation interception, increased flower buds, more regular production (Rademacher 2004, Vercammen et al. 2005, Rodríguez-Gamir et al. 2010, Carra et al. 2017), as well as the production of better quality fruit with smaller size, firmer fruit, more soluble solids content and less pre-harvest drop (Schupp and Ferree 1987, Ferree 1992, Carra et al. 2017). It has also been used by horticulturists to control plant size or vigor, as occurs in the production of bonsai plants. The regeneration of the root system following root pruning through the induction of lateral root (LR) formation has long been reported in different plant species (Thimann 1936, Van Overbeek 1939, Torrey 1950, Wightman et al. 1980, Biddington and Dearman 1984). To the best of our knowledge, the first report of root regeneration by root cutting documented aerial roots of tropical grape (*Vitis* sp.) (Zimmerman and Hitchcock 1935). Root pruning of wheat has also been reported to increase LR number and auxin content (Vysotskaya et al. 2001). Joshi et al. (2016) reported that root cutting or heat ablation of adventitious root cap enhanced *CYCLIN B1* expression in potato lateral root primordium (LRP) and suggested that this occurred through the activation of auxin signaling.

LR formation goes through three steps. First, LRPs are initiated from pairs of pericycle cells that possess developmental potential as plant stem cells. These pericycle cells are selected and directed to become LR founder cells and form LRs by both intrinsic and environmental signals (De Smet et al. 2007, Dubrovsky et al. 2008, Richter et al. 2009, Sugimoto et al. 2010). Secondly, LRPs develop from a single pericycle cell layer to a dome-shaped mature primordium, a process that can be divided into seven stages (Malamy and Benfey 1997, Petricka et al. 2012). Thirdly, LRPs emerge to become LRs by crossing the endodermis, cortex and epidermis (Vermeer et al. 2014, Vilches-Barro and Maizel 2015, von Wangenheim et al. 2016).

The phytohormone auxin plays fundamental roles in many aspects of plant growth and development, and it is a key regulator of LR development (Fukaki et al. 2007, Lavenus et al. 2013). Auxin signaling is known to be essential for LR formation (Casimiro et al. 2001, De Smet et al. 2006, Fukaki and Tasaka 2009, Lavenus et al. 2013); it begins with the degradation of a class of AUXIN/INDOLE-3-ACETIC ACID (Aux/IAA) through the TRANSPORT INHIBITOR RESPONSE1 (TIR1) auxin receptor (Dharmasiri et al. 2005, Kepinski and Leyser 2005), resulting in the activation of the AUXIN RESPONSE FACTOR (ARF) (Ulmasov et al. 1997, Nanao et al. 2014). ARF7 and ARF19 transcription factors further induce the expression of downstream target genes such as the *LATERAL ORGAN BOUNDARIES-DOMAIN/ASYMMETRIC LEAVES2-LIKE* (*LBD/ASL*) family genes *LBD16/ASL18* and *LBD29/ASL16,* and induce LR initiation at the protoxylem pole pericycle cells (Okushima et al. 2005, Okushima et al. 2007, Lee et al. 2009, Goh et al. 2012). Other ARFs also have a redundant role in LR formation. ARF6 and ARF8, which form a phylogenetic clade and have partially overlapping functions (Remington et al. 2004, Okushima et al. 2005), are the positive regulator for adventitious root formation and the determinant for LR plasticity in response to nitrogen (Gifford et al. 2008, Gutierrez et al. 2009). *AUX/IAA* gain-of-function mutants such as *massugu2/indole-3-acetic acid19* (*msg2/iaa19*) (Tatematsu et al. 2004), *crane/indole-3-acetic acid18* (*crane/iaa18*) (Uehara et al. 2008), *suppressor of hy2 mutation 2/indole-3-acetic acid3* (*shy2/iaa3*) (Tian and Reed 1999) and *solitary root/indole-3-acetic acid14* (*slr/iaa14*) (Fukaki et al. 2002) are auxin insensitive and are defective in LR formation.

Polar auxin transport (PAT), which mobilizes IAA from source to sink tissues, is facilitated by auxin influx carriers known as AUX1 and LIKE AUX1s (LAXs) and by auxin efflux carriers known as PIN-FORMEDs (PINs) and MULTIPLE DRUG RESISTANCE/P-GLYCOPROTEINs (MDR/PGPs) (Paponov et al. 2005, Blakeslee et al. 2005, Kramer and Bennett 2006, Swarup and Péret 2012). PAT, through these auxin transporters, collectively generates auxin gradients and maintains an auxin maximum, both of which are essential in LR formation and positioning (Casimiro et al. 2001, Marchant et al. 2002, Benková et al. 2003, De Smet 2012, Lavenus et al. 2013). AUX1 promotes LR formation and LAX3 promotes LR emergence (Marchant et al. 2002, Swarup et al. 2008). PIN-dependent local auxin gradients are considered to be an essential element for organ formation, and the dynamic rearrangement of PIN1 is correlated with the establishment of auxin gradients and LRP development (Benková et al. 2003). MDR/PGPs and PINs define two distinct auxin efflux systems, but can interact physically and functionally to modulate auxin efflux, create auxin gradients and regulate LR formation (Geisler et al. 2005, Lin and Wang 2005, Petrášek et al. 2006, Wu et al. 2007, Mravec et al. 2008). Consequently, the inhibition of PAT activity only by *N*-1-naphthylphthalamic acid (NPA) is sufficient to block LR initiation (Casimiro et al. 2001).

Natural auxin, IAA, is mainly synthesized in a two-step pathway from tryptophan. First, tryptophan is converted to indole-3-pyruvate (IPA) by the TRYPTOPHAN AMINOTRANSFERASE OF ARABIDOPSIS1/SHADE AVOIDANCE3 (TAA1/SAV3) family of aminotransferases; IPA is then converted to IAA by the YUCCA (YUC) family of flavin monooxygenases (Tao et al. 2008, Stepanova et al. 2008, Yamada et al. 2009, Mashiguchi et al. 2011, Won et al. 2011). Several lines of evidence have indicated that the IPA pathway is essential for auxin biosynthesis in *Arabidopsis thaliana*, and that the *YUC* family is a rate-limiting step in this pathway (Cheng et al. 2007, Stepanova et al. 2008, Zhao et al. 2001, Zhao 2012).

Although regeneration of the root system following injury is critical for the survival and fitness of sessile plants and has been generally observed and documented in a wide range of plant species, the molecular mechanisms underlying this regeneration process are poorly understood. While root pruning is typically used to refer to an agricultural technique employed on woody plants in the field, the cutting of roots under sterile conditions reflects the results of root pruning in the field. In this study, we report on the molecular mechanism of root regeneration following root cutting. We identified *YUC9* as the primary gene responsible for elevation of the IAA level by root cutting, and characterized the regulatory role of auxin biosynthesis and transport in this process.

## Results

### Root cutting induces LR formation

**Fig. 1:**
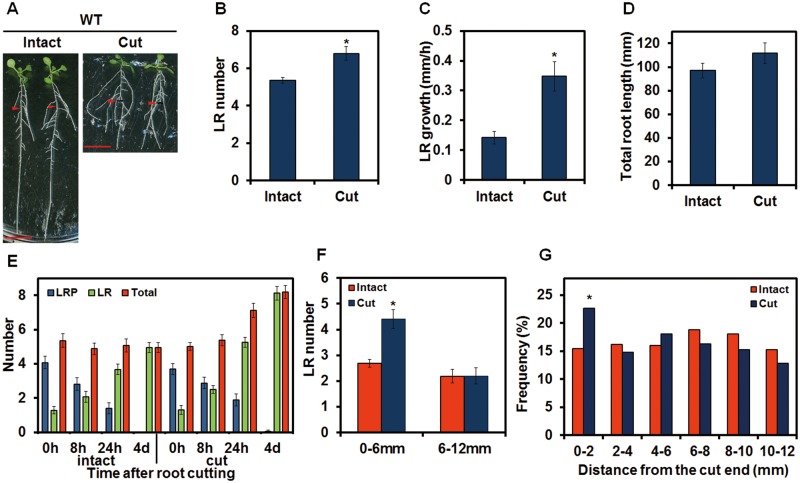
Root cutting-induced increase in lateral root (LR) number (RCN) and root cutting-induced increase in LR growth in wild-type (WT) plants. (A) Four-day-old plants were transferred to new medium and incubated for 1 d before root cutting. Photographs were taken 4 d after root cutting. Scale bars = 1 cm. Red arrowheads indicate the point 12 mm from the root–shoot junction that corresponds to the cut point. (B) The number of LRs was counted in the 12 mm area from the root–shoot junction. (C) Growth rate of the first emerged LR. (D) Total length of the root system of intact or root-cut plants 4 d after root cutting was calculated as the sum of the length of the primary root and every LR. (E) Progress of LR development after root cutting. The roots of 5-day-old plants were cut at 0 h. The number of lateral root primordia (LRPs) or LRs was counted at the indicated time points, and the sum of the LRP and LR number was defined as the total. (F) LR number within 0–6 or 6–12 mm of the cut end. (G) Histogram of LR distribution after root cutting (adventitious roots at the root–shoot junction are not included), *n* = 100. Error bars indicate the SE (*n* = 16). *Significant differences between root-cut and intact plants (Student’s *t*-test, *P* < 0.05) (B, C, F) or (χ^2^ test, *P* < 0.05) (G).

To investigate root regeneration in Arabidopsis, the primary root of 5-day-old seedlings of wild-type (WT) plants was cut at 12 mm from the root–shoot junction. The number of LRs on the remaining 12 mm long root portion in root-cut plants increased subtly but significantly after 4 d in comparison with the corresponding 12 mm long area in intact plant (Fig. 1A, B). In addition to the increase in LR number, the cut plants had longer LRs than the intact control (Fig. 1A). We measured the growth rate of the first LR proximal to the root–shoot junction in cut plants and demonstrated that it grew more than twice as fast as the intact control (Fig. 1C). Additionally, after a 4 d recovery, the total length of the root system in root-cut plants was the same as in the corresponding intact controls (Fig. 1D). We named the root-cutting induced increase in LR number RCN (root cutting-induced increase in LR number). In this study, we focus on RCN. To investigate LR initiation and LRP development during the RCN in more detail, the number of LRPs and LRs within 12 mm from the root–shoot junction was determined. In intact plants, 5-day-old seedlings (0 h in Fig. 1E) have approximately one LR and four LRPs per seedling. With incubation, the LRP number decreased and new LRs emerged (Fig. 1E). The total number of LRPs and LRs in the 12 mm area was constant from 0 h to 4 d in intact plants, indicating that few new LRPs formed after the 5-day-old seedling stage in this area (Fig. 1E). Compared with intact plants, the total number of LRPs and LRs in root-cut plants was higher than in intact plants 24 h after treatment, indicating that new LRPs initiated at 8–24 h after root cutting (Fig. 1E). At 4 d after incubation, almost all of the LRPs developed into LRs, and few LRPs remained in the 12 mm area of interest in both intact and root-cut plants (Fig. 1E). To investigate the spatial pattern of LRs and the primary root, the LR number was counted in the region proximal to the cut end (0–6 mm from the cut end) and the region distal to the cut end (6–12 mm from the cut end). LR induction was evident in the 0–6 mm area (Fig. 1F) in plants with root cutting. Furthermore, the histogram of LR distribution after root cutting showed the newly initiated LRs enriched in the 0–2 mm area from the cut end (Fig. 1G). These results indicate that RCN occurs proximal to the cut end of the root. In addition, we also observed the enhancement of adventitious root growth after root cutting. Both the number of adventitious roots at the root–shoot junction and the length of the first emerged adventitious root increased significantly following root cutting (Supplementary Fig. S1), suggesting that root cutting promotes the growth of the whole root system.

### LR-defective mutants related to auxin signaling recover LRs after root cutting

**Fig. 2:**
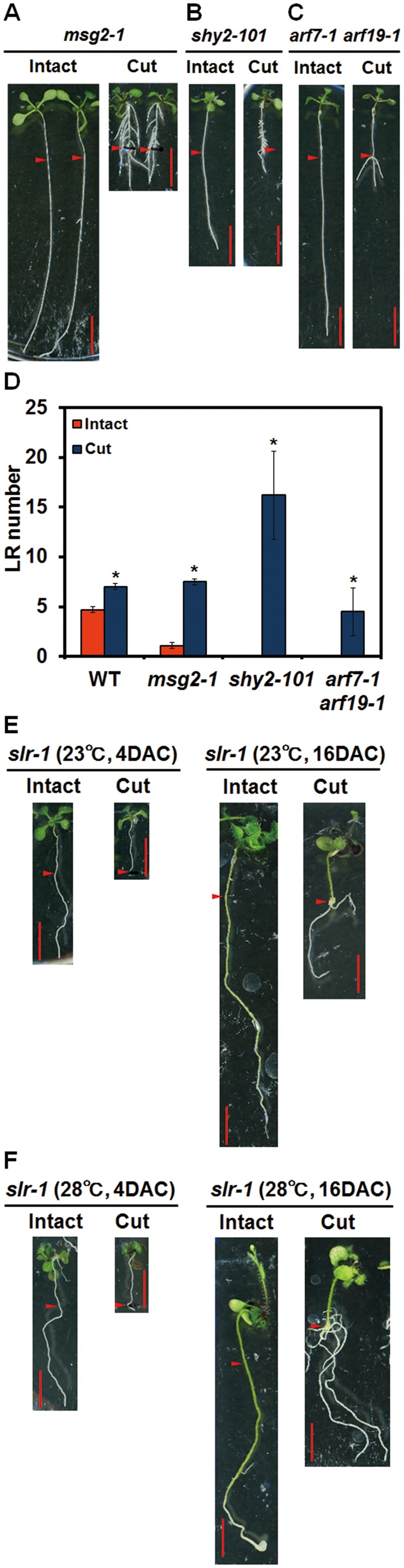
Root cutting induced LR formation in auxin-related mutants. Four-day-old plants were transferred to new medium and incubated for 1 d before root cutting. After root cutting, plants were incubated at control temperature (23°C) (A–E) or high temperature (28°C) (F). Photographs were taken at 4 (A–C, E left, F left) or 16 (E right, F right) days after root cutting (DAC). Scale bars = 1 cm. Red arrowheads indicate the point 12 mm from the root–shoot junction that corresponds to the cut point. (D) The number of LRs was counted in the 12 mm area from the root–shoot junction. Error bars indicate the SE (*n* = 16). *Significant differences between root-cut and intact plants (Student’s *t*-test, *P* < 0.05).

Auxin signaling mutants have been reported to be defective in the initiation or emergence of LRs (Tian and Reed 1999, Fukaki et al. 2002, Tatematsu et al. 2004, Okushima et al. 2005, De Smet et al. 2006, Fukaki and Tasaka 2009). As we have shown that root cutting induced the new initiation of LRs and promoted the growth of LRs in WT plants, we thus examined the RCN in dominant *AUX/IAA* mutants and loss-of-function *ARF* mutants to see how they respond to root cutting (Fig. 2; Supplementary Table S1). In the auxin signaling mutant *msg2-1*, which is a dominant mutant of *AUX/IAA19*, where the number of LRs in the intact plants was lower than in intact WT plants, LR number recovered to the WT level after root cutting (Fig. 2A, D). However, the LRs in *msg2-1* are shorter than those in the WT (Figs. 1A, 2A). It is noteworthy that *shy2-101*, the dominant mutant of *IAA3*, formed shorter LRs throughout the primary root following root cutting (Fig. 2B, D). More surprisingly, the *arf7-1 arf19-1* mutant that is defective in LR initiation (Okushima et al. 2005, Okushima et al. 2007) formed a few LRs at the cut end (Fig. 2C, D). All the examined auxin-related mutants showed RCN to a different degree, except for *slr-1* where RCN did not occur within 4 d of root cutting (Fig. 2E left). However, a longer incubation of 16 d after root cutting or exposure to high temperature (28°C) induced RCN in *slr-1* (Fig. 2E right, F), confirming a previous notion that LR was occasionally induced in *slr-1* when the primary root was cut off (Fukaki et al. 2002). Induction of RCN in these auxin mutants indicates that root cutting overcomes the defect of auxin signaling. This is also true for the induction of adventitious roots in *msg2-1* and *shy2-101* (Fig. 2; Supplementary Fig. S1).

### Root cutting activates auxin signaling

**Fig. 3:**
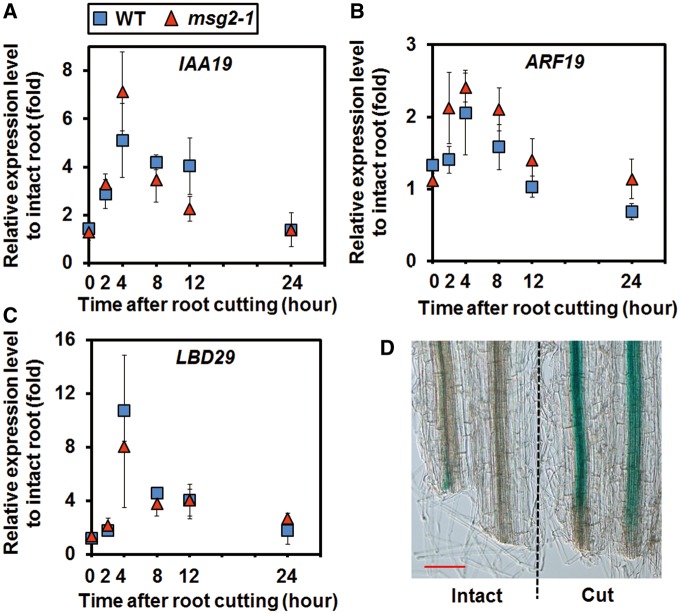
Auxin signaling genes are induced by root cutting. (A–C) Relative expression of *IAA19*, *ARF19* and *LBD29* after root cutting. Error bars indicate the SE of three independent biological replicates. (D) The expression pattern of *IAA19.* The roots of 6-day-old seedlings expressing *pIAA19*::*GUS* were cut at 12 mm from the root–shoot junction. After 6 h, β-glucuronidase (GUS) staining was performed. The expression of *pIAA19*::*GUS* was observed in the cut end of root-cut plants and the corresponding area in intact plants. Scale bar = 0.1 mm.

It was not expected that auxin signaling mutants would recover LR formation by root cutting (Fig. 2; Supplementary Table S1). As auxin signaling is essential for LR initiation and development (De Smet et al. 2006, Fukaki and Tasaka 2009), how auxin signaling is involved in RCN response was further investigated. The expression of an early auxin-inducible gene *Aux/IAA19* (Tatematsu et al. 2004), the auxin-inducible transcription factor gene *ARF19* (Okushima et al. 2005, Okushima et al. 2007) and its downstream gene *LBD29* (Lee et al. 2009) increased after root cutting (Fig. 3A–C). Expression of *Aux/IAA19* reached a peak 4 h after root cutting (Fig. 3A). Notably, activation of *IAA19* expression was evident in the cut end, as shown by β-glucuronidase (GUS) staining (Fig. 3D). LR formation-related genes *LBD16* and *LBD18* (Supplementary Fig. S2A, B), and auxin efflux carrier genes *PIN1*, *PIN3* and *PIN7* (Supplementary Fig. S2C–E), which are known to respond to auxin (Vieten et al. 2005), were also induced by root cutting. These results suggest that root cutting induces LR formation through activating the auxin signaling pathway.

### The RCN requires auxin biosynthesis and PAT activity

**Fig. 4:**
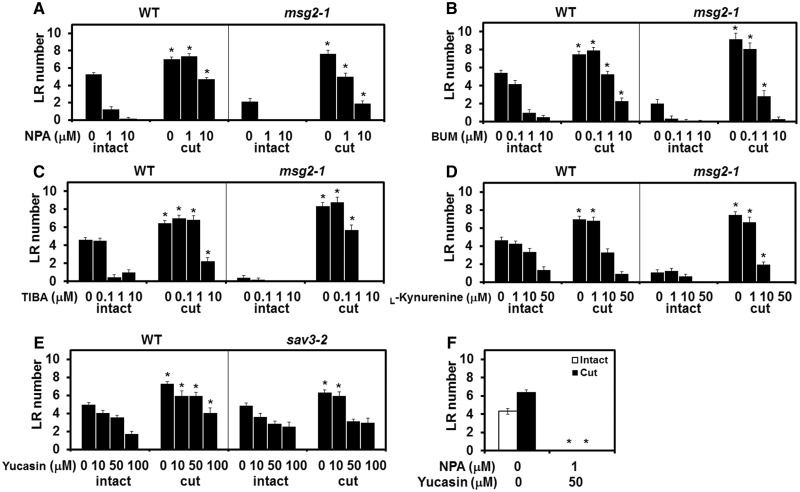
RCN is robust to auxin transport inhibitors but not to auxin biosynthesis inhibitors. Four-day-old plants were transferred to medium with or without the auxin transport inhibitors *N*-1-naphthylphthalamic acid (NPA) (A), 2-[4-(diethylamino)-2-hydroxybenzoyl]benzoic acid (BUM) (B) or 2,3,5-triiodobenzoic acid (TIBA) (C) or the auxin biosynthesis inhibitor l-kynurenine (D) and incubated for 1 d before root cutting. The number of LRs was counted in the 12 mm area from the root–shoot junction 4 d after root cutting. (E) WT and *sav3-2* plants were treated with different concentrations of yucasin. (F) The combination of yucasin and NPA abolished LR formation in both intact and cut plants. Error bars indicate the SE (*n* = 16). *Significant differences in root-cut vs. intact plants (A–E) and treated vs. control plants (F) (Student’s *t*-test, *P* < 0.05).

As auxin transport is important for the accumulation of auxin that promotes LR formation (Lavenus et al. 2013), we employed a PAT inhibitor, NPA (Fujita and Syono 1996, Casimiro et al. 2001), to study the role of auxin transport in RCN. Surprisingly, root cutting recovered LR number completely in plants treated with a moderate concentration (1 µM) of NPA and even in those treated with a higher concentration (10 µM) that completely abolished LR growth in intact plants (Fig. 4A). NPA also abolished LR formation in *msg2-1* intact plants; however, root cutting still induced LR formation (Fig. 4A). We further examined different PAT inhibitors; the MDR/PGP-specific inhibitor 2-[4-(diethylamino)-2-hydroxybenzoyl]benzoic acid (BUM) (Kim et al. 2010), and 2,3,5-triiodobenzoic acid (TIBA) (Geldner et al. 2001). Like NPA, root cutting restored LR formation under the effect of either BUM or TIBA (Fig. 4B, C). These results indicate that RCN is resistant to PAT inhibition to a certain extent. The robust nature of the RCN led us to hypothesize that auxin biosynthesis is involved in RCN. Besides auxin transport, *de novo* auxin biosynthesis is another means by which auxin accumulates (Lavenus et al. 2013). l-Kynurenine was identified as an auxin biosynthesis inhibitor that targets TAA1 and its related enzymes TAA RELATEDs (TARs) (He et al. 2011). Moderate (10 µM) to high concentrations (50 µM) of l-kynurenine abolished RCN, resulting in root-cut plants with a similar number of LRs to the intact control (Fig. 4D). Yucasin is an auxin biosynthesis inhibitor that targets YUCCA flavin-containing monooxygenase, which is downstream of TAA1/TARs (Nishimura et al. 2014). Nishimura et al. (2014) reported that the inhibitory effect of yucasin on auxin biosynthesis is limiting in WT plants but more obvious in *sav3-2*, the mutant of *TAA1.* In WT plants, the number of LRs was higher in root-cut plants in the presence of yucasin, indicating the occurrence of RCN (Fig. 4E). However, the LR number of root-cut *sav3-2* was comparable with that of intact *sav3-2* plants with high concentrations of yucasin (50 and 100 µM), indicating a defect in the RCN mechanism (Fig. 4E). These results suggest that auxin biosynthesis plays an essential role in RCN. We also attempted to block both PAT and auxin biosynthesis at the same time to determine the robustness of RCN. The combination of yucasin and NPA completely blocked LR formation in both intact and root-cut WT plants (Fig. 4F). Taken together, these results indicate that auxin biosynthesis is the primary factor for RCN, while both auxin biosynthesis and PAT activities together are necessary for the maximum RCN.

### Root cutting elevates the endogenous IAA level

**Fig. 5:**
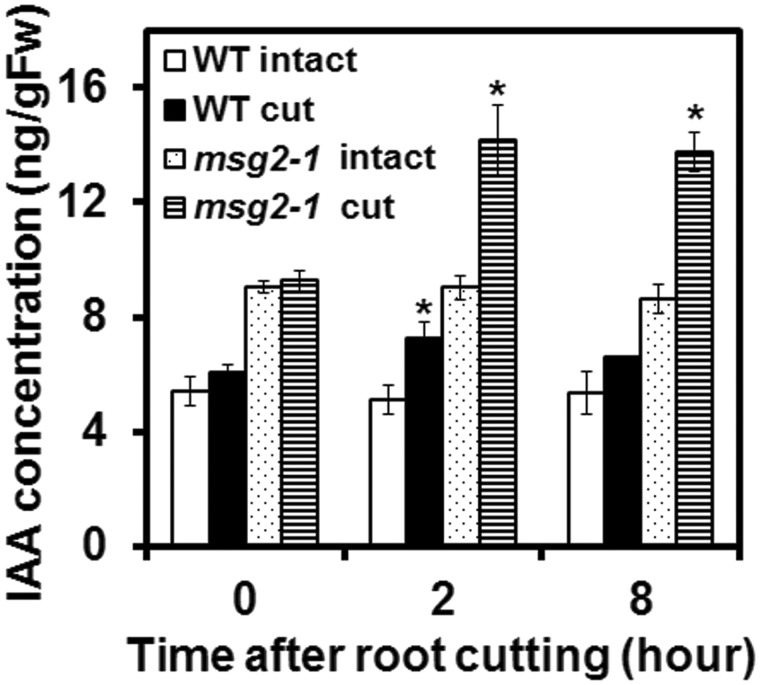
IAA is induced by root cutting. Auxin concentration was measured after root cutting at the indicated time points. Error bars indicate the SE of three independent biological replicates. *Significant differences compared with 0 h (Student’s *t*-test, *P* < 0.05).

The requirement of auxin biosynthesis for RCN led us to measure the endogenous IAA level in roots. IAA increased 2 h after root cutting in both the WT and *msg2-1* (Fig. 5), with a higher IAA level found in both intact and root-cut *msg2-1* plants than in WT plants. Thus we hypothesize that RCN is activated through the elevation of the endogenous IAA level following root cutting. To confirm this hypothesis, we applied the exogenous auxin, 1-naphthaleneacetic acid (NAA), and found that LRs were actually induced in a dose-dependent manner in both the WT and *msg2-1* (Supplementary Fig. S3). Induction of LRs in *msg2-1* suggests that elevation of the auxin level can overcome the auxin insensitivity and LR deficiency in *msg2-1.*

### YUC9 is responsible for induction of auxin biosynthesis in RCN

**Fig. 6:**
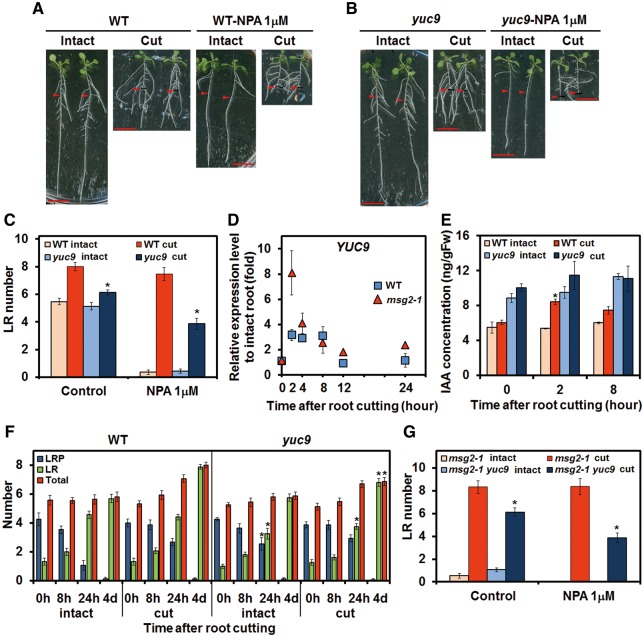
The role of *YUCCA9* (*YUC9*) following root cutting. Four-day-old WT (A) and *yuc9* (B) plants were transferred to medium with or without NPA and incubated for 1 d before root cutting. Photographs were taken 4 d after root cutting. Scale bars = 1 cm. Red arrowheads indicate the point 12 mm from the root–shoot junction that corresponds to the cut point. (C) LR number of the WT and *yuc9* following root cutting in the presence or absence of NPA. LR number was counted within 12 mm from the root–shoot junction 4 d after root cutting. (D) Relative expression level of *YUC9* after root cutting. (E) IAA level of the WT and *yuc9* at the indicated time points. (F) Progress of LR development after root cutting. The roots of 5-day-old plants were cut at 0 h. The number of LRPs or LRs was counted and the sum of LRP and LR number was defined as the total. (G) LR number of *msg2-1* and *msg2-1 yuc9* following root cutting in the presence or absence of NPA. LR number was counted within 12 mm from the root–shoot junction 4 d after root cutting. Error bars indicate the SE from 16 seedlings (C, F, G) or from three independent biological replicates (D, E). *Significant differences compared with WT plants (C, F), *msg2-1* (G) or 0 h (E) (Student’s *t*-test, *P* < 0.05).

The reduction of RCN in *sav3-2* in the presence of yucasin (Fig. 4E) suggests a major role for *YUC* genes in RCN. All available *yuc* mutants were examined and *yuc9* was found to have reduced RCN (Supplementary Fig. S4). Although LR number in the intact *yuc9* was similar to the intact WT, RCN of root-cut *yuc9* plants was reduced significantly compared with root-cut WT plants in control medium; this reduction was even more pronounced in NPA medium (Fig. 6A–C). This is consistent with previous results showing that RCN was completely abolished by a combination of NPA and yucasin (Fig. 4F). These results, in combination with previous observations that *YUC9* is expressed in root tissue (Hentrich et al. 2013, Chen et al. 2014), led us to characterize *YUC9* further. We thus studied the gene expression of *YUC9* and found that *YUC9* expression was transiently affected by root cutting, increasing 2 h after root cutting before decreasing to the basal level after 12 h (Fig. 6D). Notably, the activation of *YUC9* preceded the induction of *AUX/IAA19* by 2 h (Fig. 3A). We then examined the endogenous IAA level. Although *yuc9* had a higher level of IAA than the WT in intact plants, the level of IAA was not increased following root cutting in *yuc9* as it was in the WT (Fig. 6E). Finally, LR initiation and LRP development were examined in *yuc9*; this confirms that RCN was lower 4 d after root cutting in *yuc9* than in the WT (Fig. 6F). The expression level of *TAA1/SAV3* was also examined. Although the increase of *TAA1/SAV3* expression was not evident in whole roots (Supplementary Fig. S2F), it becomes more obvious near the cut end (2.5 mm within cut end) (Supplementary Fig. S2G); however, this induction was later than the peak of expression of *IAA19*, *ARF19*, *LBD29*, *PIN1* and *PIN7* (Fig. 3A–C; Supplementary Fig. S2C, E), suggesting that *TAA1/SAV3* plays a complementary role in RCN. Taken together, these results indicate that *YUC9* is a key gene for RCN. To confirm and provide further evidence that the RCN of *msg2-1* is also dependent on the *YUC9*-mediated pathway, the *msg2-1 yuc9* double mutant was generated. In either the presence or absence of NPA, RCN was reduced significantly in *msg2-1 yuc9* compared with *msg2-1*, indicating that the RCN in *msg2-1* is also mediated by *YUC9* (Fig. 6G). Since the phytohormone jasmonic acid (JA) has been reported to be implicated in *YUC9*-mediated auxin biosynthesis in wounded leaves in Arabidopsis (Hentrich et al. 2013), we examined the role of JA in RCN. Methyl jasmonate (MeJA) increased LR number in both intact and cut plants in the WT, but not in *yuc9* (Supplementary Fig. S5). However, induction of the JA level was not found following root cutting (data not shown).

### Redundancy of YUC family genes involved in RCN

**Fig. 7:**
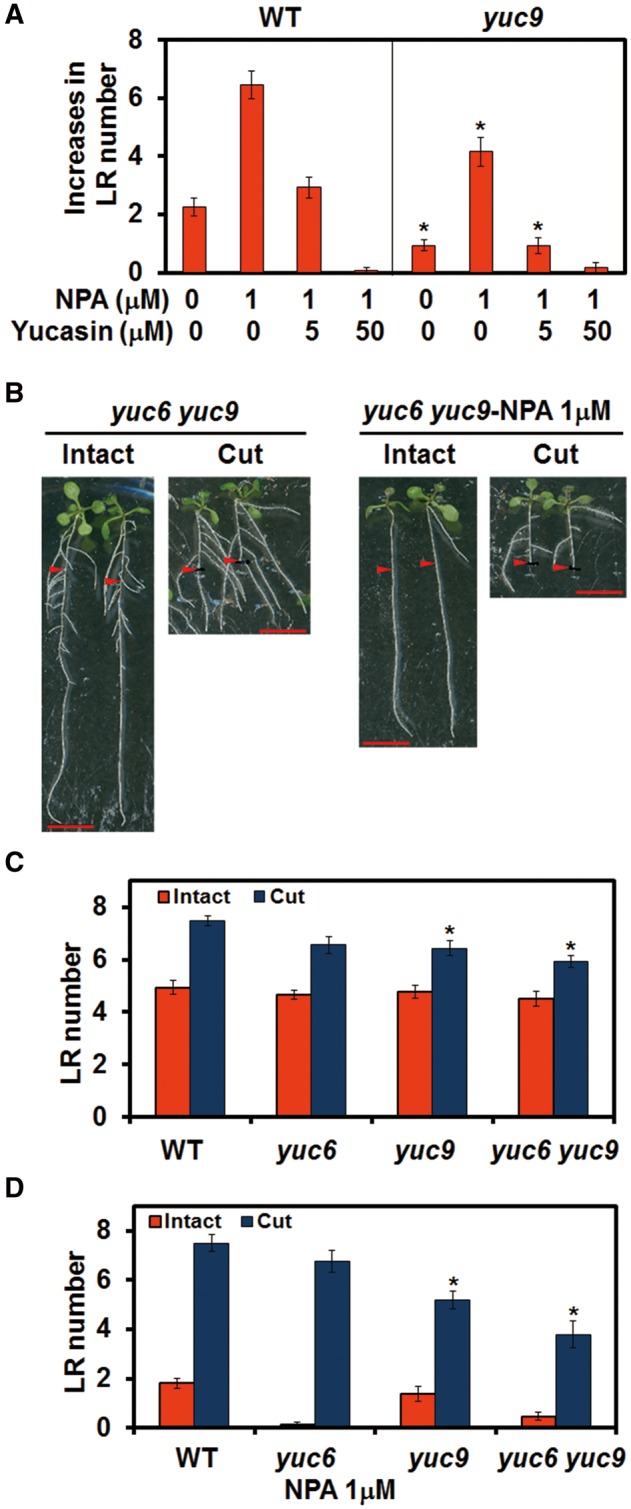
Functional redundancy of *YUC* family genes in RCN. (A) Increases in LR number under different concentration of yucasin in the presence of NPA. Increases in the LR number were calculated by subtracting the LR number of intact plants from those of root-cut plants. (B) Four-day-old plants were transferred to medium with or without NPA and incubated for 1 d before root cutting. Photographs were taken 4 d after root cutting. Scale bars = 1 cm. Red arrowheads indicate the 12 mm point from the root–shoot junction that corresponds to the cut point. LR number of plants after root cutting in the absence (C) or presence (D) of NPA. LR number was counted within 12 mm from the root–shoot junction 4 d after root cutting. Scale bars = 1 cm. Error bars indicate the SE (*n* = 16). *Significant differences compared with the WT (Student’s *t*-test, *P* < 0.01).

While there was a significant reduction in RCN in *yuc9* compared with the WT, some RCN was still observed in this mutant, suggesting that there may be functional redundancy in the *YUC* gene family. To examine this hypothesis, different concentrations of yucasin were applied in conjunction with NPA, and a further reduction of RCN in *yuc9* was observed (Fig. 7A). The sensitivity of *yuc9* to yucasin suggests that other yucasin-sensitive enzymes are involved in RCN in the *yuc9* mutant; these enzymes are likely to be members of the YUC family. A subtle reduction of RCN on *yuc6* was noticed, but did not show a significant difference compared with the WT (Fig. 7C, D). *yuc6* was introgressed into *yuc9*, and we observed further reduction of RCN in *yuc6 yuc9,* especially in the presence of NPA (Fig. 7C, D). These results confirmed the gene redundancy among the *YUC* gene family in terms of RCN.

### Synergistic effect of auxin biosynthesis and PAT activity on RCN

**Fig. 8:**
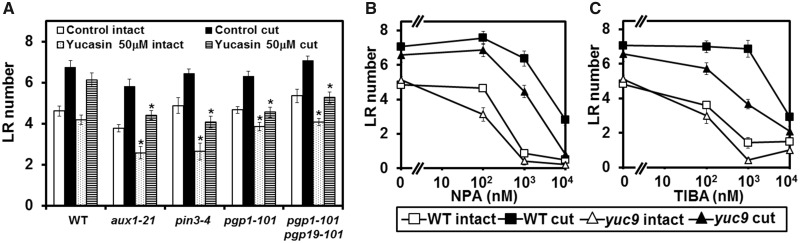
Synergystic effect of auxin biosynthesis and polar auxin transport (PAT) on RCN. (A) The number of LRs was examined in PAT-related mutants with or without yucasin treatment. Reduction of LR number in different concentration of the auxin transport inhibitors NPA (B) and TIBA (C). The number of LRs was counted in the 12 mm area from the root–shoot junction 4 d after root cutting. Error bars indicate the SE (*n* = 16). *Significant differences compared with plants in control medium (Student’s *t*-test, *P* < 0.05).

Pharmaceutical inhibition of both PAT and auxin biosynthesis influences RCN (Figs. 4F, 7A), and *yuc9* or *yuc9 yuc6* show high sensitivity to NPA (Figs. 6C, 7A, B, D). These results suggest a synergistic effect of PAT and auxin biosynthesis on RCN. We therefore examined RCN in auxin transport mutants under yucasin treatment. As in WT plants, LR number increased in the PAT-related mutants *aux1-21*, *pin3-4*, *pgp1-101* and *pgp1-101 pgp19-101* in control medium following root cutting. The number of LRs in these plants was, however, reduced in the presence of yucasin in both intact and root-cut plants. Conversely, yucasin treatment did not affect LR number in either root-cut or intact WT plants (Fig. 8A). This indicates that these mutants were more sensitive to yucasin than WT plants, and suggests that RCN requires PAT activity, especially with the restriction of auxin biosynthesis. Next, the degree of RCN in the WT and *yuc9* under NPA and TIBA treatment was further measured; here, both NPA and TIBA suppressed RCN more strongly in *yuc9* than in the WT (Fig. 8B, C), indicating that *yuc9* is more sensitive to PAT inhibitors than WT plants. These results indicate that PAT and auxin biosynthesis activity work synergistically. The deficiency of either PAT activity or auxin biosynthesis activity does not lead to an obvious phenotype following root cutting but renders the plants more susceptible to the condition when the other factor is restricted, suggesting that PAT activity or auxin biosynthesis contributes to the plants’ tolerance during cut-induced root regeneration.

## Discussion

Root pruning is a horticultural technique that is widely used in both the agricultural industry and by amateur gardeners. In this study, we investigated root cutting as a model system for root pruning. We found that root cutting promoted the regeneration of the root system by increasing the number and growth of both LRs and adventitious roots. Here we focus on the mechanism of the increase in LR number (RCN). There are three potential explanations for RCN; first, a root-cutting-induced signal directly activates the auxin signaling pathway; secondly, auxin transport is enhanced by root cutting; and thirdly, auxin biosynthesis is enhanced by root cutting. LR numbers did, however, increase in auxin signaling mutants after root cutting, contrary to the first hypothesis (Fig. 2; Supplementary Table S1). While PAT inhibitors inhibited LR formation in intact plants, root cutting was able to overcome this inhibition (Fig. 4A–C), suggesting that root cutting was able to compensate for the reduction in PAT activity by inhibitors. Abolishment of RCN by auxin biosynthesis inhibitors suggests that auxin biosynthesis is the primary factor regulating RCN (Fig. 4D, E). Furthermore, we showed that *YUC*-mediated auxin biosynthesis is responsible for RCN, and it co-operates with PAT to facilitate the maximum RCN.

### *YUC9* and *YUC* gene family members redundantly mediate cut-induced auxin biosynthesis

The YUC family of flavin monooxygenases are the enzymes for the final step of the IPA biosynthesis pathway in Arabidopsis (Won et al. 2011, Mashiguchi et al. 2011, Zhao 2012). *YUC1*, *YUC2*, *YUC4* and *YUC6* were suggested to be mainly responsible for auxin biosynthesis in shoots, while *YUC3*, *YUC5*, *YUC7* and *YUC8* are responsible for this process in roots (Won et al. 2011). Overexpression of *YUC3* gene in shoots led to an auxin overproduction phenotype in shoots, but did not change the *DR5-GUS* expression level in the root tip, and cannot rescue auxin deficiencies in the root, suggesting that shoot-produced auxin is not sufficient for root development (Chen et al. 2014). An increase in *YUC9* expression in roots suggests that auxin biosynthesis following root cutting occurs in the root (Fig. 6D). This, together with mutant analysis (Fig. 6B, C, F) and endogenous IAA quantification (Fig. 6E), suggests that *YUC9* is a key gene in RCN. Analysis of the RCN in the *msg2-1 yuc9* double mutant confirmed that the cut-induced root regeneration is also mediated by *YUC9* in *msg2-1* (Fig. 6G). In *Nicotiana attenuata* leaves, the induction of *YUC*-like genes by herbivore attack or wounding has previously been reported (Machado et al. 2016). In Arabidopsis leaves, expression of *YUC9* has been shown to increase following wounding (Hentrich et al. 2013). In Arabidopsis leaf explants, involvement of *YUC* genes in adventitious root formation was reported (Chen et al. 2016). In this study, functional redundancy of other *YUC* family members was investigated. Although only *yuc9* showed a significant reduction of RCN among *yuc* mutants in control medium, *yuc6* and *yuc7* also showed significant reduction of RCN in the presence of NPA (Supplementary Fig. S4A, B), suggesting that other members of the *YUC* family are involved in RCN. Further reduction of RCN in *yuc9* by application of yucasin (Fig. 7A) and lower RCN in the *yuc6 yuc9* double mutant than in the *yuc9* single mutant also support this hypothesis (Fig. 7B–D). These results, together with previous studies, reveal the differential roles of *YUC* family genes in different biological contexts, indicating the tissue specificity and functionally redundancy of these genes. JA is a hormone involved in wound signaling that induced *YUC9* expression in wounded leaves (Hentrich et al. 2013), and induction of LR formation by exogenous MeJA is dependent on *YUC9* (Supplementary Fig. S5). Therefore, it is quite probable that JA is the upstream signal that activates *YUC9* expression after root cutting. In Arabidopsis leaf, wounding rapidly increased the JA and JA-Ile level, with the maximum level of JA occurring at 1 h and the JA-Ile level reaching a peak at 40 min after wounding (Suza and Staswick 2008). Both the JA and JA-Ile level remained elevated for at least 8 h after wounding (Suza and Staswick 2008). In this study, the JA and JA-Ile level in root tissues 1–12 mm from the root–shoot junction was quantified at 2 and 8 h after root cutting, which corresponds to the induction time point of *YUC9* expression, and no elevation of the JA or JA-Ile level was observed (data not shown). However, local accumulation of JA near the wounding site can not to be excluded, and further investigation may be necessary.

### Root cutting modifies the developmentally controlled LR patterning

Developmentally controlled LR initiation in intact plants was suggested to start from specific pericycle cells that gain the competence to become founder cells soon after they leave the basal meristem (Dubrovsky et al. 2000, Moreno-Risueno et al. 2010). After removal of the root tip, however, we found new initiation of LRs from mature regions of the primary root (Fig. 1A, B, E) especially near the cut end (Fig. 1F, G). This new initiation represents reprogramming of pericycle cells to be LR founder cells, which involves dedifferentiation of pericycle cells and activation of cell cycles in pericycle cells (Ferreira et al. 1994, Malamy and Benfey 1997, Dubrovsky et al. 2000, Beeckman et al. 2001, Himanen et al. 2002). Auxin has been suggested to serve as a local morphogenetic trigger to specify LR founder cells (Dubrovsky et al. 2008). Exogenous auxin can reprogram pericycle cells to become LR founder cells in mature regions of the root (Blakely et al. 1988, Laskowski et al. 1995) (Supplementary Fig. S3). In this study, root cutting triggered new initiation of LRs in the WT plants (Fig. 1E). Except for *slr-1*, where division of pericycle cells is blocked during LR initiation (Fukaki et al. 2002), the auxin signaling mutants used in this study all showed RCN under standard root cutting conditions. Even in *slr-1*, RCN did occur with a longer post-cutting incubation period or with exposure to high temperatures (Fig. 2E, F), indicating the robustness of the RCN response that changes the programmed patterning of LRs. In the dominant mutant of *AUX/IAA19*, *msg2-1*, fewer LRs were generated than in the WT (Tatematsu et al. 2004); root cutting recovered LRs of *msg2-1* to a similar extent as the WT (Fig. 2A, D). Exogenous auxin also induced LRs in *msg2-1* plants (Supplementary Fig. S3). These results suggest that *msg2-1* possesses auxin sensitivity but that the sensitivity threshold has been heightened by the dominant *AUX/IAA19/MSG2* mutation such that the endogenous IAA level is not strong enough to promote LR formation. Root cutting in *msg2-1,* however, promoted IAA biosynthesis sufficiently to overcome the threshold to promote LR emergence (Fig. 4; Supplementary Fig. S3). *shy2-101*, which has an increase in the number of LR initiation sites but where LRPs remain dormant (Goh et al. 2012), formed high density but relatively shorter LRs throughout the primary root after root cutting (Fig. 2B, D), suggesting that root cutting promoted the emergence of the dormant LRPs in *shy2-101.* It is noteworthy that after root cutting, *arf7-1 arf19-1*, which is defective in the very early stage of LR initiation (Okushima et al. 2005, Okushima et al. 2007), formed LRs proximal to the cut end, suggesting that new initiation event occurred in the cut end (Fig. 2C, D). Taken together, root cutting modified the LR patterning through inducing LR initiation and promoting LR development not only in the WT plants but also in the auxin signaling mutants.

### Involvement of the root tip in RCN

Ditengou et al. (2008) reported that gravity-induced bending of the *arf7 arf19* root relocated PIN1 protein in protoxylem cells at the first stage of LR initiation. As LRs did not subsequently emerge from the *arf7 arf19* root, it has been suggested that *ARF7* and *ARF19* were not required for the first stage of LR initiation but were necessary for the later stages of LR development. Manual removal of the root tip after root bending, however, resulted in the emergence of LRs from the bending site of the *arf7 arf19* root, suggesting that there is an unknown mobile signal from the root tip which suppresses LR emergence (Ditengou et al. 2008). In the present study, root cutting alone was able to induce LR formation in *arf7-1 arf19-1* (Fig. 2C, D), suggesting that removal of the root tip suppression signal promotes both the initiation and emergence of LRs. In previous studies, the primary root tip has also been proposed to have an inhibitory effect on LR formation (Zimmerman and Hitchcock 1935, Ditengou et al. 2008). Genetic ablation of the root cap cell reduced primary root growth and increased the total number of LRs (Tsugeki and Fedoroff 1999), further suggesting the presence of a mobile signal from the root tip that suppresses LR formation. Conversely, auxin in the outer root cap cells was considered to be required for LR formation in intact plants (Van Norman 2015, Xuan et al. 2015). These contrasting observations suggest that the root tip has both a promotional and an inhibitory effect on LR formation in different contexts; the mechanism regulating this process requires further investigation.

### Auxin biosynthesis and auxin transport co-operatively regulate RCN

Auxin biosynthesis plays a critical role in RCN. *YUC9* as a primary gene responsible for RCN was induced 2 h after root cutting (Fig. 6D), preceding the induction of the auxin signaling gene *IAA19* (Fig. 3A). The inhibitory effect of PAT inhibitors was compensated by root cutting (Fig. 4A–C). This compensation is likely to have been mediated by activation of auxin biosynthesis as well as the increase in the level of PAT-related gene expression (*PIN1*, *PIN3* and *PIN7*) following root cutting (Supplementary Fig. S2C–E). Since *PIN1*, *PIN3* and *PIN7* gene expression was able to be up-regulated by auxin treatment in root (Vieten et al. 2005), we suggest that the observed increases in the expression level of these PAT-related genes resulted from the elevation of IAA level after root cutting (Supplementary Fig. S2C–E). Induction of *TAA1/SAV3* expression following that of *IAA19*, *LBD29*, *PIN1* and *PIN7* suggests that *TAA1/SAV3* plays a complementary role in cut-induced auxin biosynthesis by providing the substrate of *YUC9*, IPA (Fig. 3A, C; Supplementary Fig. S2C, E–G). Inhibition of both PAT activity and auxin biosynthesis totally abolished the RCN (Fig. 4F), indicating that they act synergistically. PAT-related mutants, *pin3-4*, *aux1-21*, *pgp1-101* and *pgp1-101 pgp19-101* showed higher sensitivity to yucasin than the WT, with LR number decreased in both intact and cut plants following yucasin treatment (Fig. 8A). The auxin biosynthesis mutant *yuc9* was more sensitive to auxin transport inhibitors than the WT (Fig. 8B, C). These results indicate that auxin transport and auxin biosynthesis work together and may compensate for each other in RCN; however, when one of them is defective, the other will become more essential and sensitive to affect RCN. Therefore, both PAT activity and auxin biosynthesis are essential for the robustness of the plants to regenerate the root system in response to root cutting.

### Model for cut-induced LR formation

**Fig. 9:**
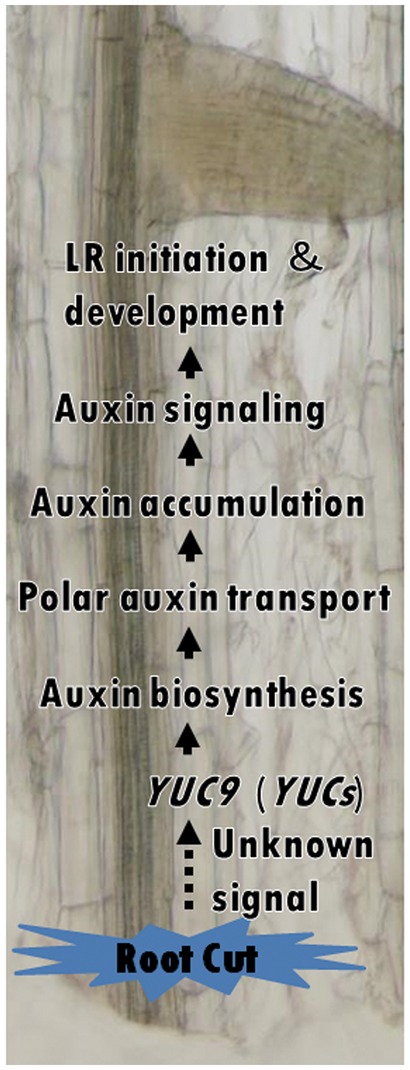
Model of the synergistic regulation of RCN by auxin biosynthesis and polar auxin transport. Root cutting activates the expression of *YUC9* and other *YUC* family genes, resulting in the elevation of the auxin level, which further induces PAT-related gene expression. Enhanced PAT activity leads to auxin accumulation and activation of downstream auxin signaling pathways which induces LR initiation and LR development.

To date, how mechanical damage such as root cutting regulates the regeneration of root systems and modifies the number and placement of LRs to form a new root system architecture is largely unknown. In this study, we provide a model for root cutting-induced auxin biosynthesis through primarily the activation of *YUC9* and other *YUC* family genes. This results in the elevation of the endogenous auxin level that induces PAT-related gene expression and enhances PAT activity before activating downstream auxin signaling genes and inducing RCN (Fig. 9). This model clarifies a previously unknown link between root cutting and *YUC9* induction. While the signal activating *YUC9* and regulating the response to root cutting is yet to be characterized, it is plausible that this signal is strongest at the cut end and spreads upward to the whole root. Further investigations are necessary to reveal the missing link between the cut-induced signal and the activation of *YUC*-mediated auxin biosynthesis.

## Materials and Methods

### Plant materials and growth conditions

*Arabidopsis thaliana* mutants and WT plants used in this study were in the Columbia background, except for *yuc5* which is in the Landsberg *erecta* background. Joanne Chory’s laboratory provided *sav3-2* (Tao et al. 2008). *yuc1*, *yuc2*, *yuc3*, *yuc4-1*, *yuc5*, *yuc6*, *yuc7*, *yuc8*, *yuc9*, *yuc10* and *yuc11* (Cheng et al. 2006, Cheng et al. 2007, Zhao 2008) mutants were obtained from the laboratory of Yunde Zhao. Seeds of *shy2-101*, *slr-1* and *crane-2* (Fukaki et al. 2002, Uehara et al. 2008, Goh et al. 2012) were obtained from the laboratory of Hidehiro Fukaki; *arf7-1 arf19-1* and *arf6-1 arf8-2* (Okushima et al. 2005) from Yoko Okushima; *pin3-4* (Friml et al. 2003) from Jiří Friml; *aux1-21* (Roman et al. 1995) from the Arabidopsis Biological Resource Center; *pgp1-101* and *pgp1-101 pgp19-101* (Nagashima et al. 2008) from Tatsuya Sakai; and *tir1-1 afb2-3* (Parry et al. 2009) from Mark Estelle. Seeds were surface sterilized with chlorine gas for at least 30 min. Seeds were suspended in 0.3% agarose and sown on half-strength Murashige and Skoog (MS) medium (Murashige and Skoog 1962; Duchefa Biochemie) supplemented with 1% (w/v) sucrose, 0.6% (w/v) gellan gum and 0.5 mM MES pH 5.8. Stratification was performed at 4°C for 2 d in the dark. Plants were grown on vertically oriented plates at 23°C under constant light conditions. Stock solutions of phytohormones and inhibitors were prepared in dimethylsulfoxide and filtered through a 0.45 µm disc filter.

### Root cutting and quantification of LRP and LR numbers

Plants were grown on vertical plates for 4 d then transferred to new half-strength MS medium with or without inhibitors or auxin. After 1 d of pre-incubation, the root was cut 12 mm from the root–shoot junction. Plant images were acquired with a flatbed scanner (GT-X980, EPSON) 4 d after root cutting. The number of emerged LRs within 12 mm from the root–shoot junction and adventitious roots at the root–shoot junction, as well as the length of the first emerged adventitious root were analyzed with ImageJ software (version 1.48, Rasband 1997–2016). To count the number of LRPs and LRs in Figs. 1E and 6F, roots were observed with a microscope [Nikon Eclipse, PlanApo X20 (NA 0.75) and PlanApo X40 (NA 0.95) objectives, Nikon instruments]. LRPs were counted between stage II and stage VII, defined according to Malamy and Benfey (1997). Stage I LRPs were not counted for this study since the optical assessment of LRPs could interfere with their differentiation. The growth rate of the first LR was measured with time-lapse imaging using a digital camera (Lumix G4, Panasonic) with time-lapse instruments; acquired images were analyzed using ImageJ. *pIAA19::GUS* plants (Kami et al. 2014) were subjected to the root cutting procedure described above, and GUS histochemical analysis was conducted as described previously (Saito et al. 2007) with the exception of fixation and incubation times (0.5 h).

### RNA isolation and quantitative reverse transcription–PCR (qRT–PCR) analysis

Root samples were harvested at 0–11 or 0–2.5 mm (Supplementary Fig. S2G) from the cut end of roots and frozen in liquid nitrogen. Total RNA was extracted and purified using a FavorPrep Plant Total RNA Mini Kit (Favorgen Biotech Corp.). cDNA was synthesized from total RNA according to the manufacturer’s instruction (ReverTra Ace qPCR RT Master Mix with gDNA Remover, Toyobo). qRT–PCR was performed in optical 96-well plates with a LightCycler 480 II system (Roche Life Science), using KOD SYBR qPCR Mix (Toyobo). Primer pairs spanning the exon–exon junction were designed using the QuantPrime program (Arvidsson et al. 2008) to avoid genomic DNA amplification, as listed in Supplementary Table S2 (Muto et al. 2007, Blacha 2009). The specificity of reactions was verified by melting curve analysis and capillary electrophoresis (Multina, Shimadzu). Standard curve analysis was used to evaluate the efficiency of the reactions. *ACTIN2* was used as an internal standard (Muto et al. 2007). The qRT–PCR program was one cycle of 98°C for 2 min, followed by 40 cycles of 98°C for 10 s, 60°C for 10 s and 68°C for 30 s. The cycle time value was determined by using the second derivative maximum method (Tichopad et al. 2003) in the LightCycler software (version 1.5, Roche Life Science). The data were analyzed using the comparative threshold cycle (Ct) method 2^−^^ΔΔ^^Ct^ (Schmittgen and Livak 2008).

### Quantification of IAA and JA

Root samples were harvested 1–12 mm from the root–shoot junction at 0, 2 and 8 h after root cutting, and frozen in liquid nitrogen. The hormone analysis was carried out as described previously (Miyamoto et al. 2016, Enomoto et al. 2017). Briefly, samples of approximately 100 mg FW were suspended in 80% (v/v) aqueous methanol with [^13^C_6_]IAA, [^2^H_2_]JA and [^13^C_6_]JA-Ile as internal standards. Samples were homogenized and the supernatant was loaded onto a Bond Elut C18 cartridge (100 mg, 3 ml; Agilent Technologies) and eluted with 80% (v/v) aqueous methanol. The concentrated samples were subjected to liquid chromatography with electrospray ionization tandem mass spectrometry (LC-ESI-MS/MS) composed of a quadrupole tandem mass spectrometer (Agilent 6460 Triple Quadrupole mass spectrometer) with an electrospray ion source and an Agilent 1200 separation module. The raw data were extracted from the MassHunter software (Agilent Technologies) and examined in Excel (Microsoft).

## Supplementary data

Supplementary data are available at PCP online.

## Funding

This work was supported by the Ministry of Education and Culture, Sports, Science, and Technology Japan [grant No. 23120501 to M.K.W.].

## Supplementary Material

Supplementary Figures and Tables

## Abbreviations

AFB: auxin-signaling F-box protein
ARF: AUXIN RESPONSE FACTOR
Aux/IAA: AUXIN/INDOLE-3-ACETIC ACID
BUM: 2-[4-(diethylamino)-2-hydroxybenzoyl]benzoic acid
*crane/iaa18*: *crane/indole-3-acetic acid18*
GUS: β-glucuronidase
IPA: indole-3-pyruvate
JA: jasmonic acid
LAX: LIKE AUX1
LBD/ASL: LATERAL ORGAN BOUNDARIES-DOMAIN/ASYMMETRIC LEAVES2-LIKE
LR: lateral root
LRP: lateral root primordium
MDR/PGP: MULTIPLE DRUG RESISTANCE/P-GLYCOPROTEIN
MeJA: methyl jasmonate
MS: Murashige and Skoog
*msg2/iaa19*: *massugu2/indole-3-acetic acid19*
NAA: 1-naphthaleneacetic acid
NPA: *N*-1-naphthylphthalamic acid
PAT: polar auxin transport
PIN: PIN-FORMED
qRT–PCR: quantitative reverse transcription–PCR
RCN: root cutting-induced increase in LR number
*shy2/iaa3*: *suppressor of hy2 mutation2/indole-3-acetic acid3*
*slr/iaa14*: *solitary root/indole-3-acetic acid14*
TAA1/SAV3: TRYPTOPHAN AMINOTRANSFERASE OF ARABIDOPSIS1/SHADE AVOIDANCE3
TAR: TAA related
TIBA: 2,3,5-triiodobenzoic acid
TIR1: TRANSPORT INHIBITOR RESPONSE1
WT: wild-type
YUC: YUCCA
