# Supplementary material for: YUCCA9-Mediated Auxin Biosynthesis and Polar Auxin Transport Synergistically Regulate Regeneration of Root Systems Following Root Cutting
Source: Plant Cell Physiol. 2017 Sep 1;58(10):1710–23. doi: 10.1093/pcp/pcx107 (PMC5921505; doi:10.1093/pcp/pcx107)
Supplement: Supplementary Figures and Tables [file pcp-2017-e-00176-file011_pcx107.pdf]

Supplementary data

Supplementary Table S1

LR number in auxin signaling mutants

Genotype	Intact plants	Root-cut plants
wild-type (WT)	4.7±0.3	7.0±0.3
tir1-1 afb2-3	2.2±0.2	4.3±0.3
arf6-1 arf8-2	1.1±0.3	8.7±0.5

LR number within the 12 mm area from the root-shoot junction was counted. Results were indicated by mean ± SE, n = 10.

Supplementary Table S2

Gene-specific primers used for qRT–PCR analysis

Gene name	Primer name	Primer Sequence (5'-3')	Reference
ACTIN2	ACTIN2-27S	5'-CGCTCTTTCTTTCCAAGCTCATA-3'	Muto et al. 2007
	ACTIN2+55AS	5'-CCATACCGGTACCATTGTCACA-3'	
IAA19	IAA19+390S	5'-CTTCGGTTTCCGTGGCATCG-3'	This study
	IAA19+521AS	5'-CATGACTCTAGAAACATCCC-3'	
ARF19	ARF19+2988S	5'-ACAGCTCGAAGATCCGCTAACC-3'	This study
	ARF19+3098AS	5'-TGCACGCAGTTCACAAACTCTTC-3'	
LBD16	LBD16+197S	5'-TCCATGATCGATGTGAAGCTGTCG-3'	This study
	LBD16+323AS	5'-TGTGATTGCAAGAAAGCCACCTG-3'	
LBD18	LBD18+274S	5'-TCCGATGCTGTCGTAACAATTTGC-3'	This study
	LBD18+390AS	5'-TTCTGCCTGTAGATTCACCACCTG-3'	
LBD29	LBD29+274S	5'-GCAAAAATCATGCTTTGTGCTGCT-3'	Blacha 2009
	LBD29+356AS	5'-TTTGCTCTCCAACAACAGGTTGTG-3'	
PIN1	PIN1+1546S	5'-GGCATGGCTATGTTCAGTCTTGGG-3'	This study
	PIN1+1661AS	5'-ACGGCAGGTCCAACGACAAATC-3'	
PIN3	PIN3+1154S	5'-AAGGCGGAAGATCTGACCAAGG-3'	This study
	PIN3+1248AS	5'-TGCTGGATGAGCTACAGCTTTG-3'	
PIN7	PIN7+1699S	5'-CGTGTGGCCATTGTTCAAGCTG-3'	This study
	PIN7+1794AS	5'-CCCTGTACTCAAGATTGCGGGATG-3'	
YUC9	YUC9+542S	5'-ATAAGTCCGGCGAGAAATTCAGAG-3'	This study
	YUC9+682AS	5'-TCGGTAAAACATGAACCGAG-3'	
TAA1/SAV3	TAA1+67S	5'-TTCGTGGTCAATCTGGATCATGG-3'	This study
	TAA1+156AS	5'-ACCACGTATCGTCACCGTACAC-3'	

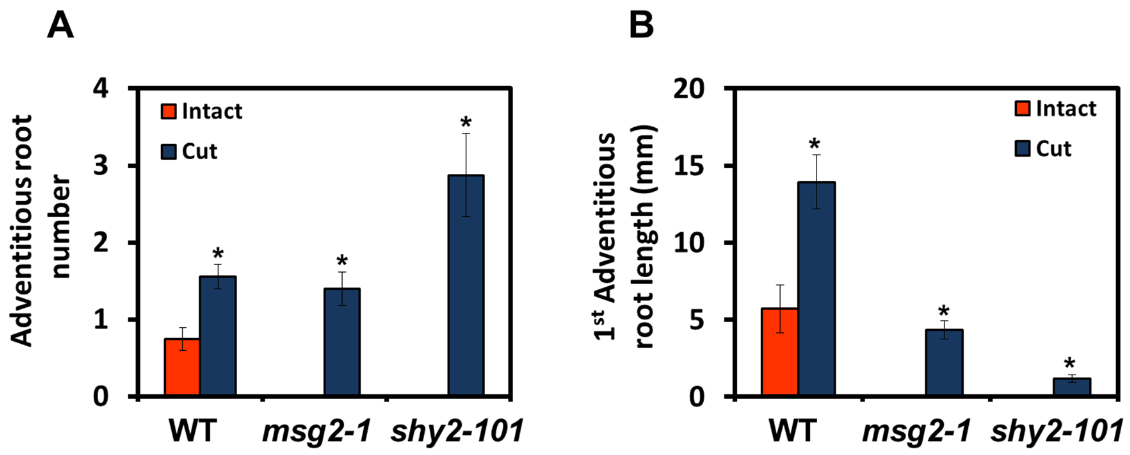

Supplementary Fig. S1. Root cutting increased adventitious root number and length. Four-day-old plants were transferred to new medium and incubated for 1 d before root cutting. The number of adventitious roots was counted (A) and the length of 1st adventitious root was measured (B) at 4 d after root cutting. Error bars indicate the SE (n=16). *Significant differences compared with intact plants (Student's *t* test, $P<0.05$).

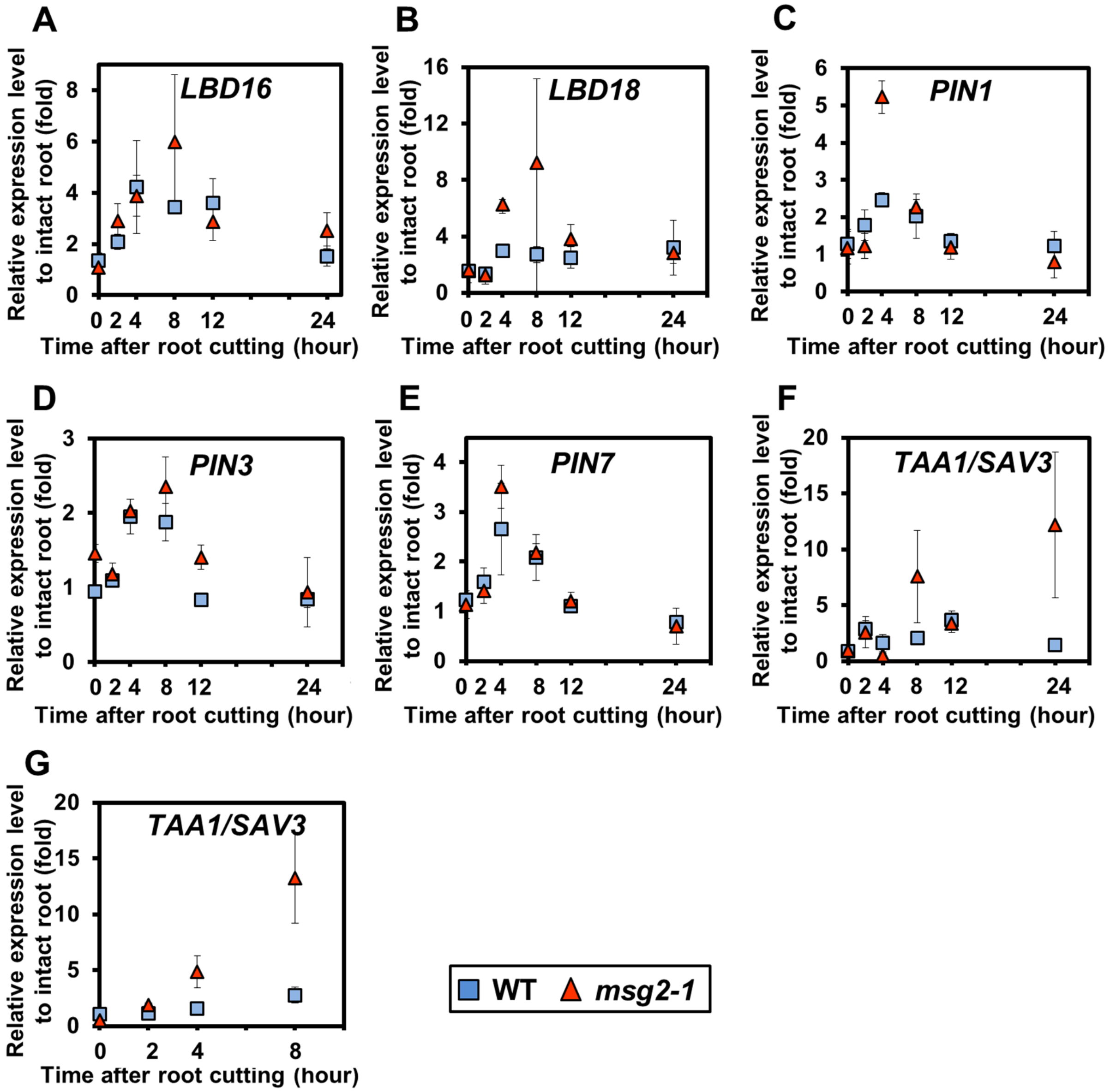

Supplementary Fig. S2. Changes of the relative expression level of LR formation-related genes (*LBD16* and *LBD18*), auxin transport genes (*PIN1*, *PIN3*, and *PIN7*), and auxin biosynthesis gene (*TAA1/SAV3*) in response to root cutting.

(A–F) RNA was extracted from 0–11 mm from the cut end. (G) RNA was extracted from 0–2.5 mm from the cut end. Error bars indicate SE from three independent biological replicates.

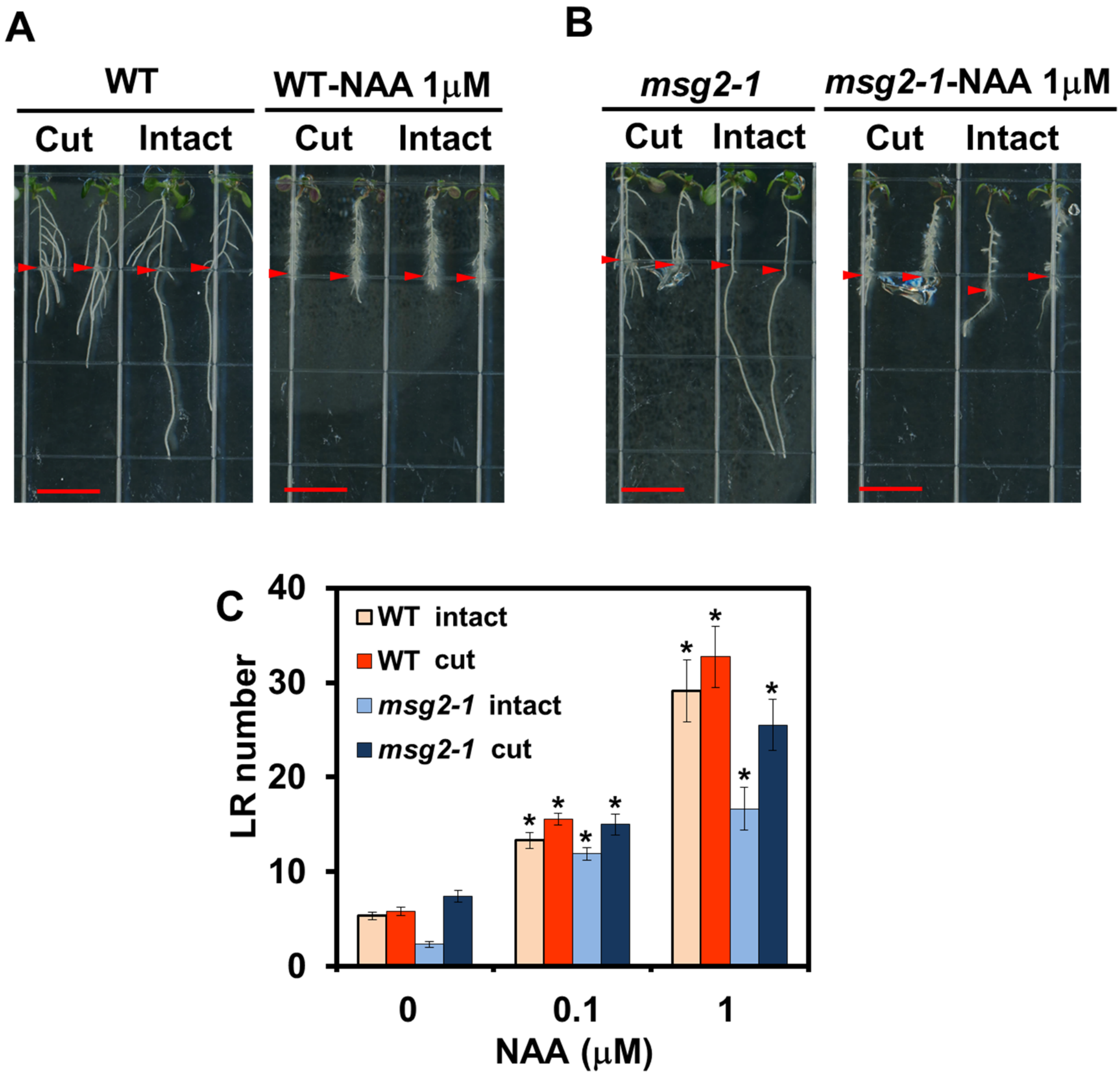

Supplementary Fig. S3. LR number of WT and *msg2-1* plants was induced by 1-naphthaleneacetic acid (NAA).

(A) Four-day-old plants were transferred to medium with or without NAA and incubated for 1 d before root cutting. Photographs were taken 4 d after root cutting. Scale bars = 1 cm. Red arrowheads indicate the point 12 mm from the root-shoot junction where the root was cut. (B) The number of LRs was counted within the 12 mm area from the root-shoot junction 4 d after root cutting. Error bars indicate the SE (n=20). *Significant differences compared with plants in control medium (Student's *t* test, $P<0.05$).

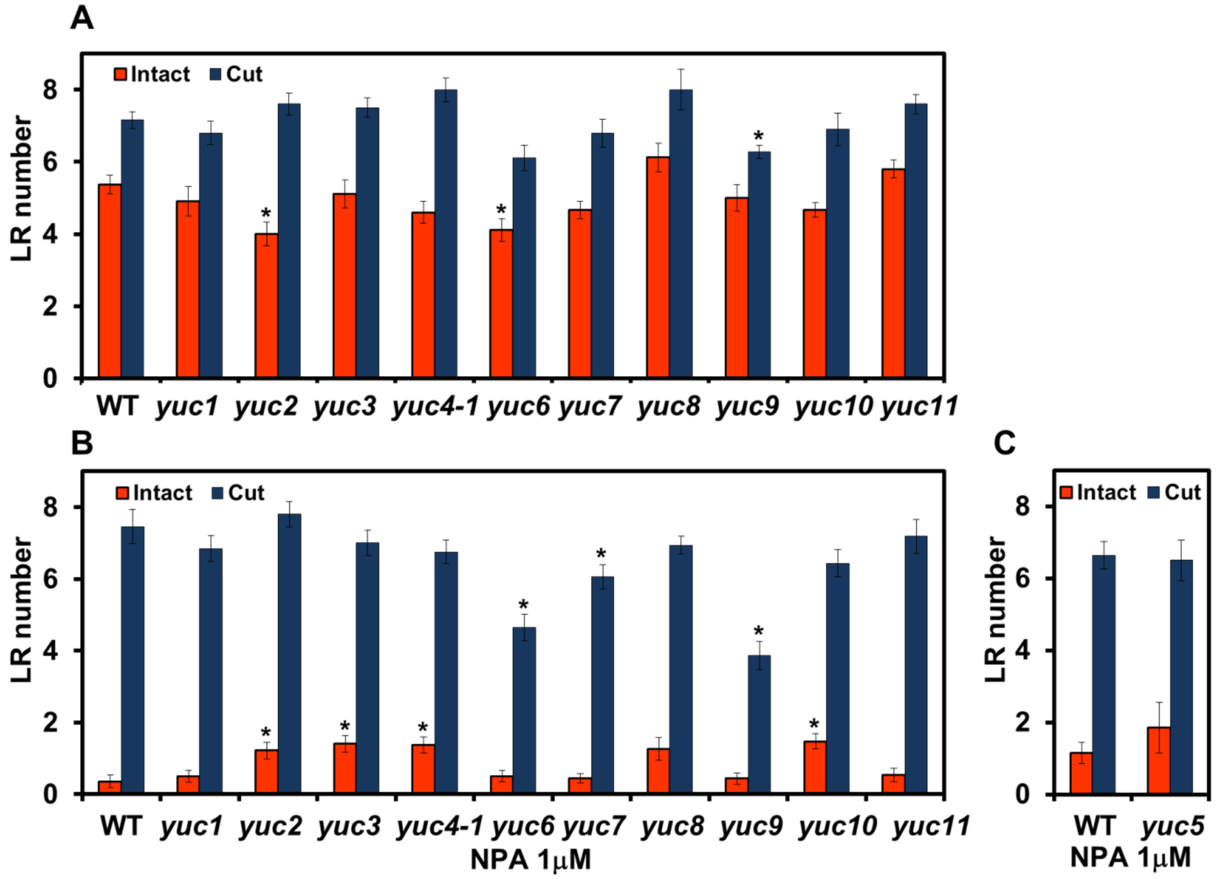

Supplementary Fig. S4. Analysis of root-cutting induced increase in LR number (RCN) in *yucca* (*yuc*) mutants.

Four-day-old plants were transferred to medium without (A) or with (B, C) N-1-naphthylphthalamic acid (NPA) and incubated for 1 d before root cutting. The number of LRs was counted within the 12 mm area from the root-shoot junction 4 d after root cutting. Error bars indicate the SE (n=16). *Significant differences compared with WT (Student's *t* test, $P<0.01$). (C) Plants were in Landsberg *erecta* background.

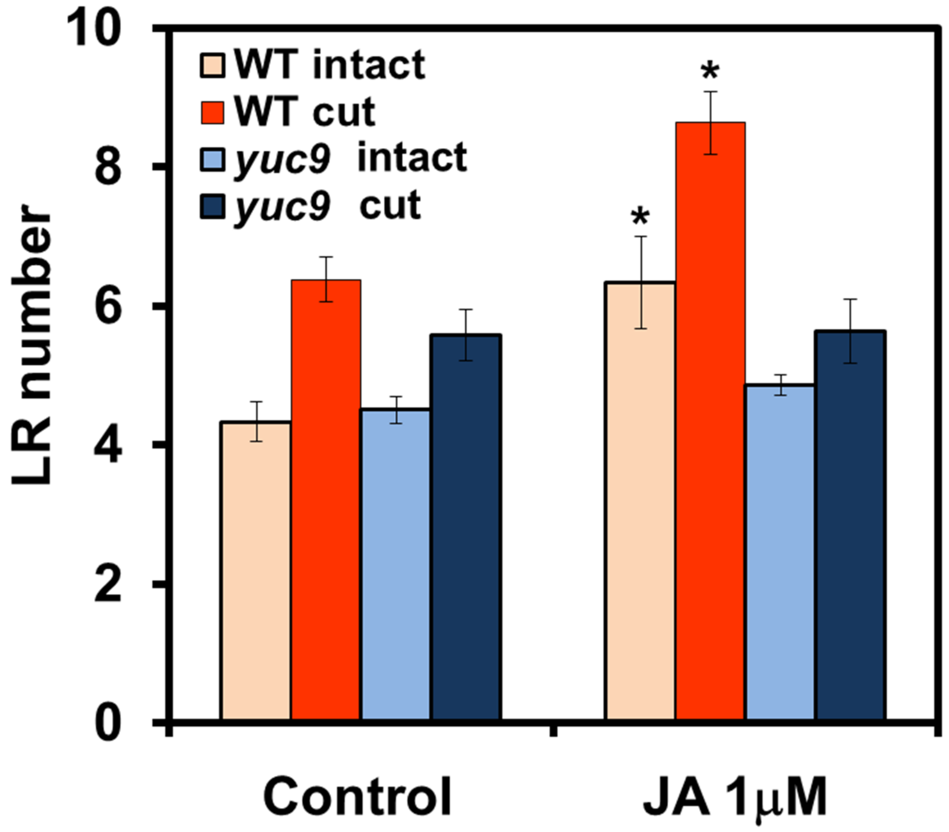

Supplementary Fig. S5. Jasmonic acid (JA) induced LR formation in WT but not in *yuc9*.

Four-day-old plants were transferred to medium with or without JA and incubated for 1 d before root cutting. The number of LRs was counted in the 12 mm area from the root-shoot junction 4 d after root cutting. Error bars indicate the SE (n=16). *Significant differences compared with plants in control medium (Student's *t* test, $P<0.05$).

References

Blacha, A.M. (2009) Investigating the role of regulatory genes in heterosis for superior growth and biomass production in *Arabidopsis thaliana*. *Ph.D. thesis*, University Potsdam, Potsdam.

Muto, H., Watahiki, M.K., Nakamoto, D., Kinjo, M. and Yamamoto, K.T. (2007) Specificity and similarity of functions of the *Aux/IAA* genes in auxin signaling of Arabidopsis revealed by promoter-exchange experiments among *MSG2/IAA19*, *AXR2/IAA7*, and *SLR/IAA14*. *Plant physiol*. 144: 187–196.
